# Reproduction of *Varroa destructor* depends on well-timed host cell recapping and seasonal patterns

**DOI:** 10.1038/s41598-023-49688-9

**Published:** 2023-12-18

**Authors:** Martin Gabel, Ricarda Scheiner, Ingolf Steffan-Dewenter, Ralph Büchler

**Affiliations:** 1 Landesbetrieb Landwirtschaft Hessen, Bee Institute Kirchhain, Erlenstraße 9, 35274 Kirchhain, Germany; 2 Department of Behavioral Physiology and Sociobiology, Biocenter, University of Würzburg, 97074 Würzburg, Germany; 3 Department of Animal Ecology and Tropical Biology, Biocenter, University of Würzburg, 97074 Würzburg, Germany

**Keywords:** Animal behaviour, Entomology, Behavioural ecology, Population dynamics

## Abstract

Resistance traits of honeybees (*Apis mellifera*) against their major parasite *Varroa destructor* have fascinated scientists and breeders for long. Nevertheless, the mechanisms underlying resistance are still largely unknown. The same applies to possible interactions between host behaviours, mite reproduction and seasonal differences. Two resistance traits, reproductive failure of mites and recapping of brood cells, are of particular interest. High rates of recapping at the colony level were found to correspond with low reproductive success of mites. However, the direct effect of recapping on mite reproduction is still controversial and both traits seem to be very variable in their expression. Thus, a deeper knowledge of both, the effect of recapping on mite reproduction and the seasonal differences in the expression of these traits is urgently needed. To shed light on this host-parasite interaction, we investigated recapping and mite reproduction in full-grown colonies naturally infested with *V. destructor*. Measurements were repeated five times per year over the course of 3 years. The reproductive success of mites as well as the recapping frequency clearly followed seasonal patterns. Thereby, reproductive failure of mites at the cell level was constantly increased in case of recapping. Interestingly, this did not apply to the occurrence of infertile mites. In line with this, recapping activity in fertile cells was most frequent in brood ages in which mite offspring would be expected. Our results suggest that mite offspring is the main target of recapping. This, in turn, leads to a significantly reduced reproductive success of the parasite.

## Introduction

Resistance to *Varroa destructor* (hereafter referred to as *Varroa*) in honeybees is broadly described as the long-term survival of bee colonies without human treatment in a given habitat^1–3^. In this, the term comprises more detailed definitions of resistance (the host’s ability to limit parasite burden) and tolerance (the host’s ability to limit the harm caused) used for individual animals^4^ and the ability to cope with various other environmental factors at the colony level. This became particularly evident when resistant honeybees were introduced into a foreign environment. There, their ability to overcome *Varroa* could no longer be observed^5–8^. The same applied to locally adapted mite-susceptible honeybees showing longer survival durations compared to foreign stock before they ultimately died from varroosis^5,8^. Resistance in honeybees therefore reflects a composition of various host-parasite interactions tuned to the respective environment^9,10^, thereby increasing the duration of survival under the given conditions.

Various *Varroa*-resistance traits (i.e., traits that lower parasite burden) frequently co-occur in the same colony^10,11^. This displays a key feature of social immunity in honeybees^12^ and fosters co-evolution from both sides of the host-parasite interaction^13,14^. Such host-parasite interactions form an equilibrium of bee and mite survival in several resistant honeybee populations^3,9,10^. However, the mechanisms behind this adaptation, i.e., the ultimate survival of colonies, can differ sharply^9,10^. Two distinct resistance traits have frequently been described as key mechanisms in surviving populations^3,9–12^: the uncapping and subsequent recapping of sealed brood cells (recapping, REC) and the reproductive failure of mites (mite non-reproduction, MNR^10^, or suppressed mite reproduction, SMR sensu lato).

MNR describes any form of reproductive failure and thus comprises mother mites with either I) no offspring (infertile), II) only female offspring (missing male) or III) progeny which is too young to reach maturity before the host cell is expected to hatch (delayed reproduction)^15^. The different forms of MNR (infertile, no male or delayed), have been less intensively studied than MNR per se^16^. Yet, their contribution to the reproductive failure of *Varroa* (MNR) can differ considerably between populations^16^ and thus likely reflects different background mechanisms.

REC was described to occur more frequently in naturally surviving colonies compared to susceptible ones^3,10,12^, while low levels were even found in *Varroa-naïve* populations^17^. It thus seems to be a specific adaptation of basal brood hygiene behaviours to the parasite. However, the role of REC as stand-alone resistance trait or proxy for removal of infested brood cells (*Varroa*-sensitive hygiene, VSH) is still under debate^10,12,18,19^. If REC decreased the reproductive success of mites on its own, it could be much more cost-effective for the honeybee host than VSH, because no brood cells need to be sacrificed^12^. While this evolutionary cost saving seems to be obvious, the true benefit of REC as resistance trait for the colony appears to be largely unknown.

MNR and REC have thus gained increasing attention in studies on the biological basis of host-parasite interactions in honeybees. Their implementation as selection criteria in resistance breeding schemes^16,20^, has led to a consensus on the need of a broader investigation of these traits^21^.

The brood investigation required for this is tedious^15^ and the accuracy of MNR and REC values strongly depends on sample size^22^. Since MNR seems to be the outcome of different background mechanisms^10^, it shows a low phenotypic repeatability compared to REC and other resistance traits^22–24^. However, these changes might simply be linked to seasonal differences in the expression of underlying behaviours (e.g., VSH or REC) due to changing nectar flows^25^, brood rearing activity^26^ or other unknown factors. Up to now, such possible effects of seasonal variation on MNR remain largely unclear. The same applies to seasonal variation of REC and its effect on mite reproduction^10,12,18,19^.

Since the set of resistance traits seems to be evolutionary tailored to the respective environment, their importance for the colony likely varies not only spatially but also temporally with external factors. The diversity of resistance traits found in naturally selected honeybee populations^9,10^ thus might also display an adaptation to temporally changing conditions.

We investigated the reproductive success of *Varroa* and the occurrence of REC in 15 consecutive trials covering three beekeeping seasons (20, 20 and 15 colonies each) to shed light on possible seasonal variations in the behaviour of mites and bees.

We thereby directly linked REC at the brood cell level (> 4100 single-infested cells) to different forms of failure in mite reproduction to gain insight into the interaction of host and parasite. In addition to the measurements at the seasonal scale, we investigated the temporal occurrence of REC and brood termination (i.e., the lethal removal of brood by worker bees) during the capped brood stage. Therefore, nearly 116,000 age-defined cells were examined using a novel image-based approach.

## Results

### Reproductive success of mites is lower in recapped cells

**Figure 1:**
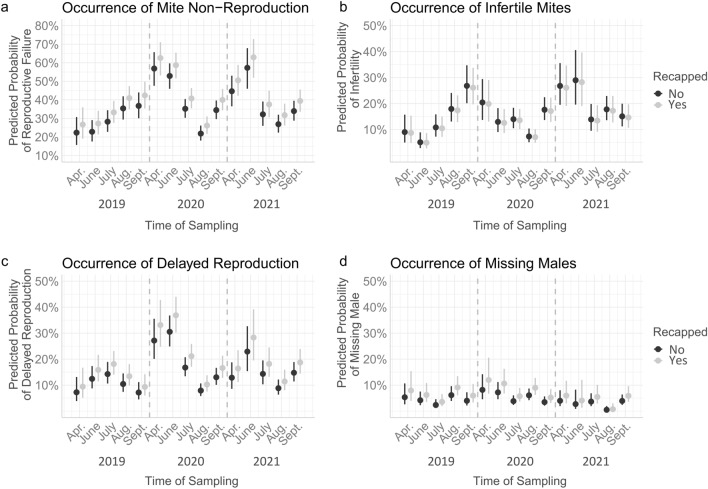
Predicted probabilities of (**a**) reproductive failure (MNR), as well as (**b**) infertility, (**c**) delayed reproduction and (**d**) missing males as cause for MNR (displayed with 95% CI). Vertical dashed lines separate consecutive years. Test statistics are given in Table 1, post-hoc comparisons are denoted in the supplementary material Table 4–7.

The probability of MNR was significantly increased in recapped cells compared to untouched cells (χ^2^ = 10.33, df = 1, *p* = 0.001, Table 1). This general pattern was displayed on all sampling dates (Fig. 1a).

**Table 1:** Model output for factors affecting the reproductive success of mites and the recapping behaviour of bees.

Dependent	Parameter	n	df	Χ^2^	*p*
Non-reproductive cells (MNR)	Recapping	4106 single-infested cells (45 colonies in 3 years)	1	10.33	0.001
	Sampling date		14	152.23	< 0.001
Infertile	Recapping		1	0.13	0.715
	Sampling date		14	94.7	< 0.001
Delayed	Recapping		1	9.15	0.003
	Sampling date		14	120.91	< 0.001
No male	Recapping		1	8.1	0.004
	Sampling date		14	32.22	0.004
Recapping	Sampling date		14	335.5	< 0.001
	Reproductive state		3	18.03	< 0.001

GLMMS of the binomial family were fitted to the data using the above-given parameters and dependent variables as well as colony and year as random factors.

When investigating the underlying cause of reproductive failure, the occurrence of delayed reproduction was also significantly increased in recapped cells (χ^2^ = 9.15, df = 1, *p* = 0.003, Table 1, Fig. 1c). Likewise, male offspring was missing more often in recapped cells (χ^2^ = 8.10, df = 1, *p* = 0.004, Table 1, Fig. 1d).

Notably, the occurrence of infertile mites did not differ between recapped and untouched cells throughout all sampling points (χ^2^ = 0.13, df = 1, *p* = 0.72, Table 1, Fig. 1b).

### Recapping frequency differs between reproductive states

**Figure 2:**
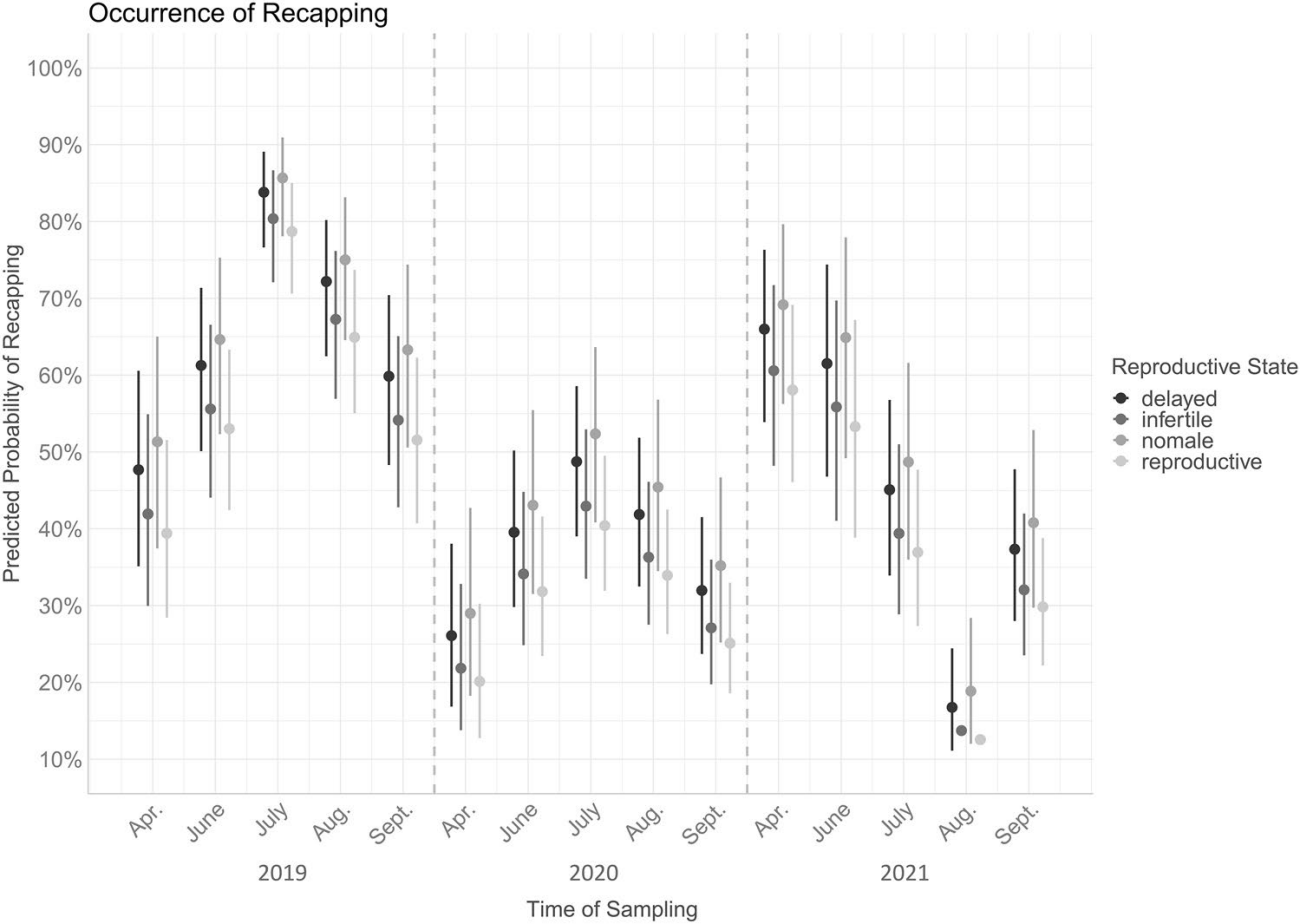
Predicted probabilities of recapping in single-infested cells (displayed with 95% CI). Vertical dashed lines separate consecutive years. Test statistics are given in Table 1, post-hoc comparisons are given in Table 2 and the supplementary material Table 8.

The probability of REC differed significantly between brood cells with different reproductive states of *Varroa* mites (χ^2^ = 18.03, df = 3, *p* < 0.001, Table 1, Fig. 2). Recapping frequency was higher in non-reproductive cells (i.e., cells with infertile mothers, delayed reproduction or missing males, n = 1480; 45.2%) compared to reproductive cells (n = 2626; 40.78%) over all single-infested cells (n = 4106, *p* < 0.001, Table 2). This held true when cells with delayed reproduction (n = 629) or missing males (n = 213) were compared to reproductive cells respectively (47.54%, *p* = 0.005 and 53.52%; *p* = 0.011, Tab. 2). REC was observed in 40.13% of infertile cells (n = 638) which did not differ from the frequency in reproductive cells (Table 2). Among the non-reproductive cells, recapping frequency did not differ between the individual causes of failure (Table 2).

**Table 2:** Pairwise comparisons of recapping frequency in single-infested cells with different reproductive states.

Comparison	Estimate	Z	*p*
Non-reproductive—reproductive	− **0.265**	− **3.510**	** < 0.001**
Infertile—reproductive	0.104	1.000	0.75
Delayed—reproductive	**0.338**	**3.322**	**0.005**
No male—reproductive	**0.482**	**3.073**	**0.011**
Infertile—no male	− 0.378	− 2.133	0.143
Delayed—infertile	0.234	1.808	0.27
Delayed—no male	− 0.144	− 0.823	0.844

Factors denoted in bold indicate significant differences between groups (*p* < 0.05; Tukey-Method adjusted for comparing 4 estimates and averaged over sampling time in case of cause comparisons).

### Mite reproduction follows seasonal patterns

The occurrence of MNR strongly differed between different sampling dates throughout the season (χ^2^ = 152.23, df = 14, *p* < 0.001, Table 1). While the probability of MNR increased steadily from April to September in 2019, it showed different patterns in 2020 and 2021 (Fig. 1a). In the latter years, failed reproduction was most frequently found between April and June, while it was least frequently observed at the end of August and beginning of September, respectively (Fig. 1a, supplementary material Table 4). This seasonal pattern was characterized by significantly higher probabilities of reproductive failure early in the season compared to mid-season brood cycles (Fig. 1a, supplementary material Table 4). The occurrence of each of the three causes for MNR was also significantly affected by the time of the season (Table 1, Fig. 1b–d).

### Recapping frequency follows seasonal patterns

Overall, occurrence of REC differed significantly between sampling dates (χ^2^ = 335.5, df = 14, *p* < 0.001, Fig. 2, Table 1). In 2019 and 2020, infested cells tended to be recapped more frequently in mid-season, while in 2021 this occurred more frequently in spring (Fig. 2, supplementary material Table 8).

### Colony level factors

MNR-Values and brood infestation showed a slightly negative correlation at the colony level (r(136) = − 0.19, *p* = 0.03, Table 3). In turn, positive correlations were found between REC of all cells investigated (RECall) and brood infestation (r(133) = 0.47, *p* < 0.01, Table 3), as well as between RECall and bee infestation (r(133) = 0.43, *p* < 0.01, Table 3). No such correlation was found between RECinf (i.e., REC of infested cells) and any of the infestation measurements (Table 3).

**Table 3:** Correlations (Spearman) between colony level factors.

	RECall	RECinf	Brood-infestation	Bee-infestation	Proportion of infertile mites	Termination rate	Image-based REC
MNR	r(133) = − .05, *p* = .58	r(135) = .04,*p* = .61	**r(136) = − .19, *p* = .03**	r(136) = − .07,*p* = .39	r(136) = − .03,*p* = .71	r(134) = .00,*p* = .98	r(134) = − .01, *p* = .94
RECall		**r(133) = .74, *p* < .01**	**r(133) = .47, *p* < .01**	**r(133) = .43,*p* < .01**	r(133) = − .01, *p* = .89	**r(131) = .44, *p* < .01**	**r(131) = .49, *p* < .01**
RECinf			r(135) = − .06, *p* = .47	r(135) = − .07, *p* = .40	**r(136) = − .21, *p* = .01**	**r(133) = .02, *p* = .02**	**r(133) = .27, *p* < .01**
Brood-infestation				**r(135) = .47, *p* < .01**	r(135) = .06, *p* = .48	**r(134) = .44, *p* < .01**	**r(134) = .35, *p* < .01**
Bee-infestation					r(136) = .10, *p* = .26	**r(134) = .47, *p* < .01**	**r(134) = .31, *p* < .01**
Proportion of infertile mites						r(134) = .01, *p* = .89	r(134) = .03, *p* = .71
Termination-rate							**r(134) = .33, *p* < .01**

Brood samples with less than 25 single-infested cells were excluded from calculations. Significant correlations are denoted in bold.

There was a positive correlation between image-based REC values and RECinf (r(133) = 0.27, *p* < 0.01) and RECall (r(131) = 0.49, *p* < 0.01) values derived from classical brood analysis (Table 3). The same applied to image-based REC values and brood infestation (r(134) = 0.35, *p* < 0.01) and bee infestation (r(134) = 0.31, *p* < 0.01, Table 3). Brood termination rates were likewise correlated with RECinf (r(133) = 0.2, *p* = 0.02) and RECall (r(131) = 0.44, *p* < 0.01), as well as bee (r(134) = 0.47, *p* < 0.01) and brood infestation (r(134) = 0.44, *p* < 0.01, Table 3). Brood termination rates and image-based REC were also positively correlated (r(134) = 0.33, *p* < 0.01, Table 3).

### Frequency of recapping and cell termination differs between brood ages

**Figure 3:**
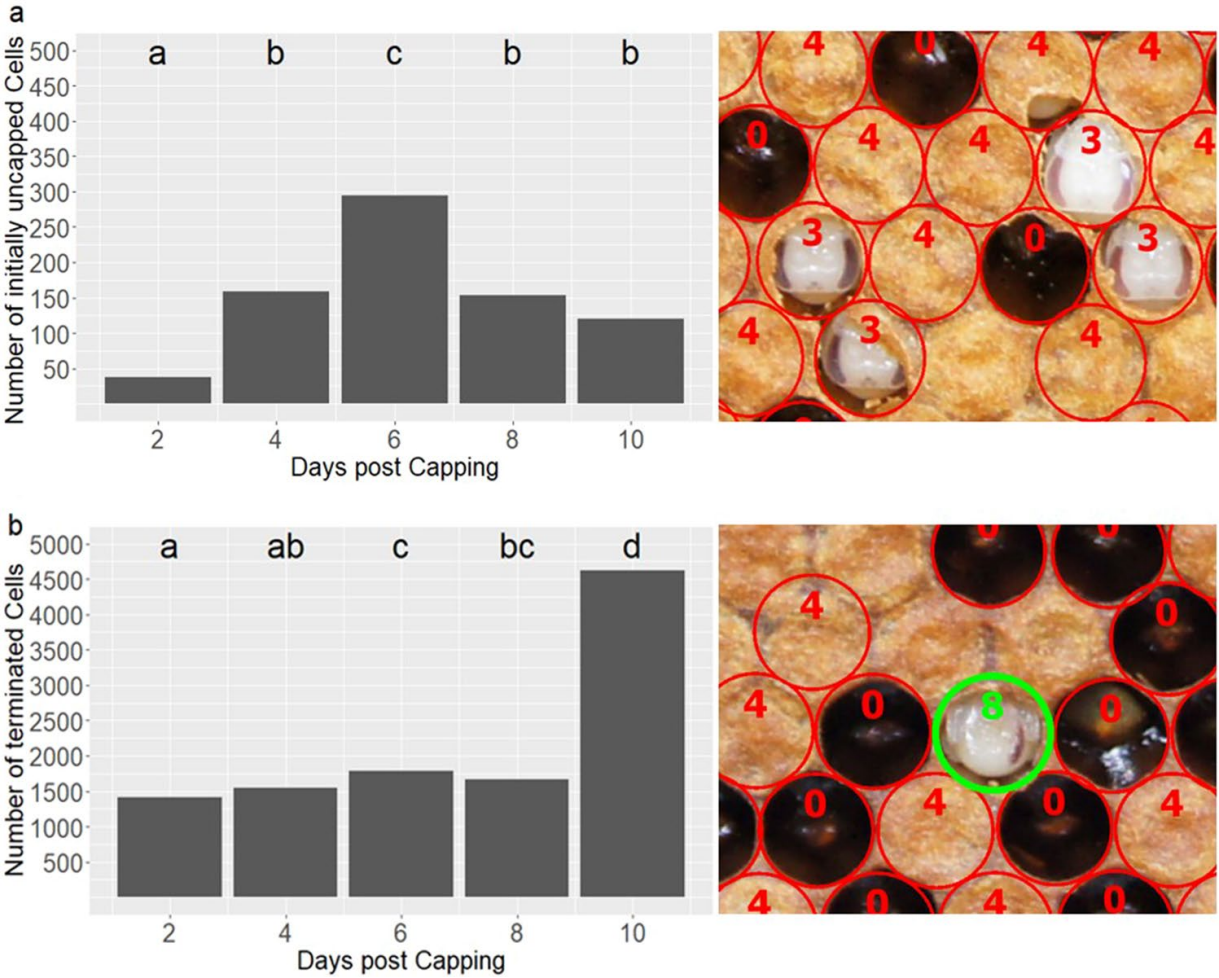
Time of (**a**) initial uncapping before REC and (**b**) cell termination with pictures taken during image-based brood investigation of (**a**) uncapped cells encoded with “3” and (**b**) terminated cell encoded with “8”. The cell codes “0” and “4” refer to empty and sealed brood cells, respectively. Brood age (days post capping) had a significant effect on the time of initial uncapping (*GLMM:* χ^2^ = 238.13, df = 4, *p* < 0.001) and the time of brood termination (*GLMM:* χ^2^ = 3783.6, df = 4, *p* < 0.001). Different letters indicate significant differences between brood ages (Tukey-Method adjusted for comparing 5 estimates, *p* < 0.05 each).

In total, 115,943 age defined cells were investigated, of which 104,898 cells (90.47%) developed normally (i.e., were not terminated). Frequency of brood cell termination differed significantly between brood ages (χ^2^ = 3783.6, df = 4, *p* < 0.001). Distinctively more cells were found empty at day 10 post capping compared to younger brood stages (*p* < 0.005, each, Fig. 3b). Cells terminated after initial recapping were excluded from recapping analysis. Recapping was observed in 764 cells, of which 609 cells showed a single recapping event and 155 cells were recorded uncapped on two or more days. Only 28 multiply recapped cells were recorded sealed in between. For the remaining multiply recapped cells it is unclear whether they were sealed between pictures or remained uncapped (“bald brood”) for longer periods. Recapping activity differed significantly between brood ages (χ^2^ = 238.13, df = 4, *p* < 0.001). Comparing all days, it was lowest at day two post capping (*p* < 0.005, each) and most frequently found six days post capping (*p* < 0.001, each, Fig. 3a).

## Discussion

Our results clearly show that *Varroa* reproduction was significantly reduced in naturally recapped brood cells. Although REC was frequently described as an important resistance trait^3,10,11,27^, beneficial effects for the host seem to be highly variable. At the colony level, high rates of REC were found to decrease *Varroa* reproduction in some cases^28,29^, while this could not be confirmed in others^17,23^. At the cell level, the results were likewise variable: effects on MNR were mainly shown for artificially uncapped cells^12^, while either no effect was found in naturally recapped cells^12,18,26^ or results differed between sample sets^24^. Thus, it was proposed that the effect of REC may sometimes be overshadowed by other mechanisms^18,26^. This would also explain contradicting reports on the relationship between REC and infestation measures at the colony level^18,23,24,29,30^. Accordingly, we observed no correlation between RECinf and infestation measures or RECinf and MNR at the colony level (Table 3), although MNR was increased in the case of REC at the cell level (Fig. 1a, Table 1).

At the host colony level, however, beneficial effects have been indicated by a slight negative correlation between MNR and brood infestation (Table 3). This supports earlier reports on increased MNR values and lower infestation levels in surviving populations^1,3,10,27^.

Thus, our findings support the formerly described diffuse effects of REC at the colony level but highlight its directly suppressing effects on mite reproduction at the cell level.

REC holds the potential to disrupt different parts of the reproductive cycle of mites from the onset of egg laying to the mating of mature offspring^28^. By discriminating the different causes of reproductive failure, our results suggest that REC mainly affects fertile mites (i.e., mites with offspring). The proportions of missing males and delayed developing female offspring were significantly increased in recapped cells (Fig. 1a,d). This was also supported by results of the image-based brood analysis: Recapping mainly occurred after the first *Varroa* offspring should have hatched in fertile cells (Fig. 3a), i.e., four and six days post capping for male and female eggs, respectively^31–33^. This contradicts previous results in which the proportion of REC increased as pupal development progressed^17^. However, these findings were based on classical brood investigations and thus could not be adjusted for the accumulation of signs of REC (i.e., holes in the pupal cocoon) over time. In other words, older brood cells were per se more likely to show signs of REC, because bees had more time to express the behaviour. Thus, the time of initial REC cannot be reconstructed in classical brood investigations. The image-based investigation presented here reveals a more accurate impression of the timing of this host behaviour, which apparently depends on the ontogenesis of the parasite.

Such a targeting of fertile mites has been frequently discussed for REC^12^ and the closely related behaviour VSH^17,34–36^ but results appeared inconsistent among studies^19,37^. In the present study, the temporal link between the occurrence of uncapping and the suspected presence of mite offspring was less prominent in terminated cells than in recapped cells (Fig. 3a,b). However, cell termination may also be triggered by other causes, e.g., developmental abnormalities that mask such temporal patterns in *Varroa*-related brood termination. Notably, the sharp increase in empty cells 10 days post capping (Fig. 3b) was most probably an effect of faster development of some worker bees and the inaccuracy of approximately one day in the age definition method used. Thus, the timing of brood termination fits the timing of recapping and the ontogenesis of *Varroa* as discussed above.

Although it remains unclear which of the cells accounted by picture trials were actually infested by mites, cell termination rates correlated with VSH in earlier studies^38^. Termination rates in our dataset were correlated with bee and brood infestation as well as REC measurements at the colony level (Table 3), supporting these earlier findings^38^. Therefore in some cases, termination of initially uncapped cells may be a second step in a complex detection cascade leading to VSH as suggested before^17,39^. Nevertheless, cells being recapped instead of terminated after initial uncapping also showed significantly increased MNR values (Table 1). REC thus appears to work as a stand-alone resistance trait in other cases, underlining the complexity and redundancy of *Varroa*-resistance mechanisms.

In the latter case, our results point towards an effect of REC on the first two descendants, which are key players for successful reproduction. The first egg (male) is mostly laid in the forward cell section near the cap. Here it is better protected from the movements of the host larva^31^. This cell section, however, is especially exposed to disturbance by worker bees opening the cell lid (REC). Eggs laid near the cell lid are thus at risk to be removed by adult bees, as was recently shown for artificially inserted items^39^. Oviposition in the anterior part of the brood cell is also common for the second egg^31^, which develops into the female with the best chances to reach maturity^33^. As^31^ observed, these protonymphs are greatly challenged by crossing the legs of the host pupae towards the feeding side and are thus moving around “hyperactive” in the anterior cell section. Likewise to disturbance of sensitive eggs, bees opening the cell lid in this phase could thus also affect mobile protonymphs, e.g., because the mite offspring goes astray through the cell opening. Although following daughter mites do not face such in-cell-migration problems^31^, a loss of the first daughter or the male would mostly be sufficient to prevent reproduction at the cell level because I) the remaining daughters would be too young to reach maturity in time (delayed reproduction) or II) adult daughters would miss a male for mating (no male). The loss of progeny would therefore explain the increased levels of delayed reproduction and missing males found after REC in this study (Fig. 1c,d). It also fits to earlier reports of decreased fecundity, i.e., the number of viable offspring in recapped cells^24^.

In addition to the precision of targeted recapping^10,12,28^, the exact timing thus seems to be crucial for the effectiveness of this resistance trait. This might also explain the results of^28^, which found a lower number of daughter mites in colonies with enhanced REC. However, this pattern only held true in surviving colonies when mite-surviving and mite-susceptible colonies were analysed separately^28^. Therefore, REC seems to be beneficial in general but survivor populations might display a better timing of the behaviour which would reflect a key point of host-parasite-adaptation. We suggest further studies to focus on the exact timing of this resistance trait to unravel the effects of REC on fertile mites. In contrast to the commonly used brood investigation method^15^, the detailed image-based approach of REC-measurements described here would better suit the needs of such studies. In turn, the standard method for MNR and REC measurements^15^ is less laborious and thus seems to be more appropriate for large-scale investigations of breeding stocks and study populations.

In contrast to fertile mites, the occurrence of infertile mothers was not related to REC at the cell level (Table 1), although the respective proportions were slightly negative correlated at the colony level (Table 3). In line with this, the lowest REC rates were found in infertile cells (40.13%) compared to reproductive cells (40.78%) and non-reproductive cells caused by delayed reproduction (47.54%) or missing males (53.52%). This additionaly supports our assumption that fertile mites (i.e., mites with offspring) are the main target of REC activity as discussed above. At the same time, it seems to be uncommon for mother mites to invade uncapped cells since this would lead to increased infertility due to mismatching host brood signals^37,40^. Also, the previously supposed^24^ mother mite emigration during uncapped brood periods seems to occur very rarely after natural infestation, since hardly any abandoned cells with mite faeces or orphan families were found. However, such emigration or removal events have been reported for cells artificially infested with mites deriving from the dispersal phase^39^.

Over all, REC seems to affect mite offspring rather than mother mites in naturally infested cells.

Although independent of REC, the occurrence of mites without offspring (i.e., infertile mothers) strongly varied throughout the seasons rather than representing a stable base line (Fig. 1b). This supports the hypothesis that mite infertility is linked to other behaviours like selective VSH^19,34^ which might in turn follow seasonal variations^25,26^. Such temporal effects are known for several resistance traits and the corresponding infestation levels^25,26,41^. The expression of REC by the bee host and MNR by its parasite was likewise variable throughout our study period (Figs. 1, 2). This seasonal variation likely reflects a change in factors both inside and outside the colony: External factors such as changing nectar flows can alter resistance behaviours by shifting work force capacities^25^. The same applies to in-hive-factors like brood rearing^26,42^ which again depend on the seasonality of the habitat. Changes in humidity and temperature could likewise have affected reproductive success, especially when combined with REC activity^43^. Notably, the pattern of reproductive success over the seasons 2020 and 2021 resemble earlier findings of^44,45^, while the MNR expression in 2019 differed from this trend for unknown reasons. The MNR patterns in 2020 and 2021 might be explained by the changes of summer and winter bees^44,45^, as well as brood breaks in winter time^26,42^. Thus, differences in brood rearing activity during the winter 2018/2019 and corresponding differences in worker longevity might also have led to the steady increase of MNR over the season 2019. However, neither the extend of brood rearing, nor the weather data was investigated in the present study and thus explanations for the differing seasonal patterns remain a subject of speculation. Nevertheless, the seasonal pattern reflects the dynamic character of host and parasite behaviours and underlines the challenges of comparable data acquisition. Although the expression of traits might often follow the patterns found in 2020 and 2021, as well as 1988 and 1989^44,45^, the pattern of 2019 and the inter-season variation between months suggest that both temporal and spatial factors need to be accounted when comparing MNR and REC data of different colonies.

In practical bee breeding, this holds major importance for performance testing and targeted selection towards increased *Varroa*-resistance. The resistance traits MNR and REC were both found to be heritable and thus selectable, if the selection methods account for variability induced by outer effects^20^. The present results suggest that MNR and REC display valuable traits for resistance breeding although targeted selection might be greatly challenged by seasonal variation. This needs to be considered in performance testing and selection schemes, e.g., by using standardized methods and appropriate analyses of test data^20^.

Our results prove that recapping behaviour of the host and mite reproduction are subject to considerable seasonal variation. Despite this overall variation at the seasonal level, the parasite's reproductive success was constantly decreased in recapped cells. In this, increased shares of delayed reproduction and missing males were linked to REC at the cell level. REC thus holds the potential as a stand-alone resistance trait but seems to add up to other mechanisms causing infertility and overall seasonal variation.

MNR and REC therefore appear to be valuable candidate traits for targeted selection towards increased *Varroa*-resistance. However, their temporal variation and other external factors need to be considered whenever measuring the expression of these traits.

## Methods

### Experimental setup

The study was conducted between 2019 and 2021 at the Bee Institute Kirchhain (Landesbetrieb Landwirtschaft Hessen, Hesse, Germany). The full-grown colonies derived from the Institute’s Carniolan breeding stock. In 2019 and 2020, 20 colonies were investigated, while 15 colonies were examined in 2021. All samples were gained at the same apiary. Colonies were uniformly re-queened with young queens after the last sampling of the respective season. At the same time, oxalic acid was applied as late summer treatment against *Varroa*. Except of the sampling of brood and bees, no *Varroa*-treatments or swarm prevention measures were applied during the study season. All hives were managed uniformly according to the local beekeeping practice but did not receive winter treatments against *Varroa*.

### Data collection

Colonies were sampled five times over the course of each beekeeping season (i.e., annually from April to September) at approximately monthly intervals as follows.

### Picture trials and sampling of brood combs

One comb with predominantly L5 larvae was chosen per colony to obtain brood of similar age. The brood comb was marked, photographed from both sides and returned to the brood chamber. Afterwards, another six consecutive pictures were taken at two days intervals up to day 12 after the first picture (i.e., approximately 10 days after capping). Thus, the picture dates partly overlapped with the intervals given by^46^ (see supplementary material Table 9). To ensure equal photo quality, the combs were mounted in a shaded box with a fixed distance of approximately 75 cm to the camera (Sony SLT-A33 with lens SAL1855, Sony Corp., Tokyo, Japan and Nikon D7500 with lens AF-S DX NIKKOR 18–300 mm, Nikon Corp., Tokyo, Japan). After the last picture of each trial (picture 7 of the respective comb, approximately 10 days after capping) brood combs were sampled and stored at − 20 °C until further brood investigation. Bee samples for standard infestation measurements were taken at the beginning of each picture trial^47^. Colonies with previous brood interruptions (e.g., due to swarming tendencies or queen change) were excluded from further analysis.

### Brood investigation

The investigation of recapping and reproductive failure of mites was over all performed according to the RNSBB protocol^15^. Yet, for colony level factors, the minimum sample size per comb was reduced to 25 single-infested cells, due to low infestation levels early in the season. Brood combs were investigated using a stereo microscope (S9i, Leica Microsystems, Wetzlar, Germany) with ten-to-30-fold magnification. The reproduction of mites was classified depending on the respective brood age as either I) successful (i.e., normal amount and age of offspring), II) infertile (i.e., no offspring at all), III) no male (i.e., only female offspring of the right age) or IV) delayed (i.e., progeny too young to reach maturity before host cell hatch). Recapping at the colony level was calculated as the proportion of recapped cells on the total number of investigated cells, i.e., infested and uninfested (RECall), and the number of infested cells only (RECinf).

### Image-based brood investigation

**Figure 4:**
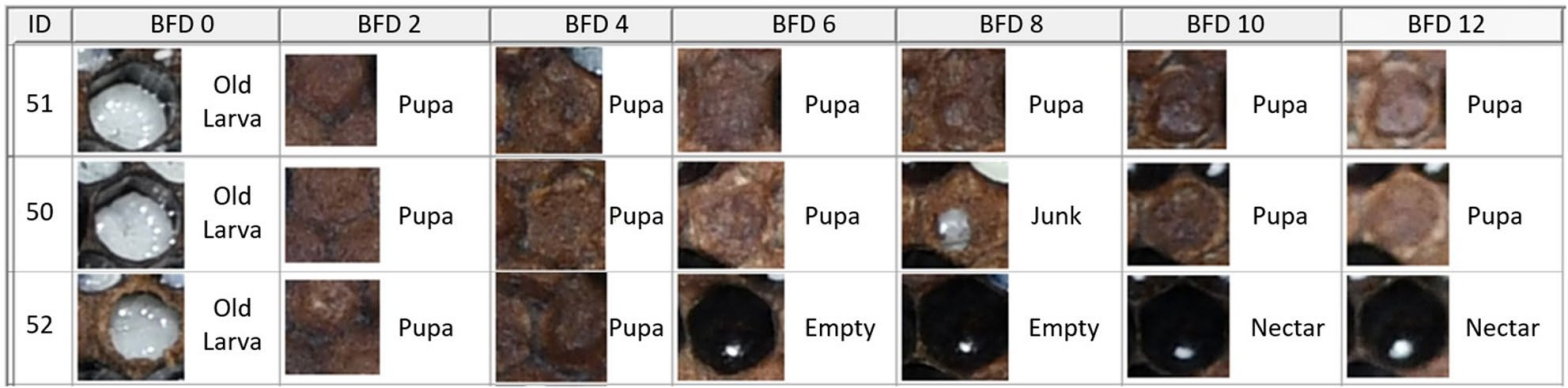
Picture trials of cell wise brood development starting approximately one day before capping (brood fixation day (BFD) 0, picture 1) up to approximately one day before emergence (BFD12, picture 7) in two days intervals (see supplementary material Table 9). Cell ID51: normal development, cell ID50: recapping at BFD8, cell ID52: brood termination at BFD6. Note that capped brood cells are always identified as “Pupa” although pupation is not completed at BFD 2 (picture 2). Uncapped brood cells are classified as “Junk” due to given category names predefined by the software.

Brood development was accounted cell wise based on the picture trials. Only age defined brood cells (L5 on picture 1 and sealed on picture 2) were used for further investigation (n = 115,943 cells). Alignment of pictures, cell determination and brood classification was performed using the software HiveAnalyzer (Version 2.33, Visionalytics, Pleidelsheim, Germany). All automatic steps of picture alignment and brood classification^48^ were individually checked and manually corrected if needed. This especially applies for uncapped brood cells (“bald brood”) which could not be identified automatically (Figs. 3a, 4). Uncapped cells which were sealed in a following picture were counted as recapped (Fig. 4). Brood cells which were uncapped and sealed several times were accounted in a separate category (multiply recapped). Cells which showed unusual development (i.e., any other cell content than sealed or uncapped brood after picture 2) were counted as terminated (Figs. 3b, 4).

### Statistical analyses

The R environment (version 4.1.0, R Core Team 2021) was used for statistical analyses. Generalized linear mixed-effect models (glmer) from the binomial family (logit) were conducted to estimate the probabilities of recapping and different forms of non-reproduction at the cell level^49^. The occurrence of recapped cells and non-reproductive mites (including different types of failure) was considered as response variable. Time (i.e., date of sampling) and recapping status or reproductive state of the cell were implemented as fixed explanatory variables. Individual colonies were included as a random factor. The same applied for beekeeping seasons to account for sample clusters within each year. Day post capping was implemented as fixed variable for image-based recordings of the first recapping event and brood termination. In this case, colony and individual cell were used as random factors. The DHARMa package^50^ was used to account for residuals and over-dispersion. Tukey-post-hoc tests (emmeans package^51^) were performed as subsequent pairwise comparisons among factor levels. For colony level measurements, spearman rank correlations were calculated using the psych package^52^. Samples with less than 25 single-infested cells were excluded from these analyses.

### Supplementary Information

Supplementary Tables.

## Data Availability

The datasets generated during and/or analysed during the current study are available from the corresponding author on reasonable request.
