# Supplementary material for: Reproduction of Varroa destructor depends on well-timed host cell recapping and seasonal patterns
Source: Sci Rep. 2023 Dec 18;13:22484. doi: 10.1038/s41598-023-49688-9 (PMC10728205; doi:10.1038/s41598-023-49688-9)
Supplement: Supplementary file 1 — Supplementary Tables. [file 41598_2023_49688_MOESM1_ESM.pdf]

Supplementary Information

Reproduction of *Varroa destructor* depends on well-timed host cell recapping and seasonal patterns

Authors: Martin Gabel[1,2*], Ricarda Scheiner[2], Ingolf Steffan-Dewenter[3] & Ralph Büchler[1]

[1]Landesbetrieb Landwirtschaft Hessen, Bee Institute Kirchhain, Erlenstraße 9, 35274 Kirchhain, Germany

[2]Department of Behavioral Physiology and Sociobiology, Biocenter, University of Würzburg, 97074 Würzburg, Germany

[3]Department of Animal Ecology and Tropical Biology, Biocenter, University of Würzburg, 97074 Würzburg, Germany

*Corresponding author, Mail: MartinSebastian.Gabel@llh.hessen.de

Supplementary Information Table 4: Pairwise comparisons of the occurrence of MNR in single infested cells over the study period. Factors denoted in bold indicate significant differences between groups (p< 0.05; Tukey-Method adjusted for comparing 15 estimates and averaged over recapping status of cells).

Contrast	Estimate	Z	p
April 2019 - June 2019	-0.029	-0.109	1.000
April 2019 - July 2019	-0.314	-1.246	0.996
April 2019 - August 2019	-0.645	-2.593	0.375
April 2019 - September 2019	-0.705	-2.730	0.286
**April 2019 - April 2020**	**-1.524**	**-5.344**	**<0.001**
**April 2019 - June 2020**	**-1.362**	**-5.401**	**<0.001**

April 2019 - July 2020	-0.637	-2.679	0.318
April 2019 - August 2020	0.028	0.117	1.000
April 2019 - September 2020	-0.605	-2.520	0.426
**April 2019 - May 2021**	**-1.032**	**-3.780**	**0.013**
**April 2019 - June 2021**	**-1.540**	**-4.900**	**<0.001**
April 2019 - July 2021	-0.502	-1.907	0.850
April 2019 - August 2021	-0.246	-0.989	1.000
April 2019 - September 2021	-0.581	-2.370	0.538
June 2019 - July 2019	-0.285	-1.420	0.986
June 2019 - August 2019	-0.617	-3.118	0.112
June 2019 - September 2019	-0.676	-3.221	0.084
**June 2019 - April 2020**	**-1.495**	**-6.092**	**<0.001**
**June 2019 - June 2020**	**-1.333**	**-6.459**	**<0.001**
June 2019 - July 2020	-0.609	-3.258	0.075
June 2019 - August 2020	0.057	0.296	1.000

June 2019 - September 2020	-0.576	-3.040	0.138
**June 2019 - May 2021**	**-1.003**	**-4.345**	**0.001**
**June 2019 - June 2021**	**-1.511**	**-5.422**	**<0.001**
June 2019 - July 2021	-0.473	-2.153	0.698
June 2019 - August 2021	-0.218	-1.067	0.999
June 2019 - September 2021	-0.553	-2.775	0.260
July 2019 - August 2019	-0.332	-1.881	0.863
July 2019 - September 2019	-0.391	-2.057	0.764
**July 2019 - April 2020**	**-1.210**	**-5.256**	**<0.001**
**July 2019 - June 2020**	**-1.048**	**-5.518**	**<0.001**
July 2019 - July 2020	-0.324	-1.944	0.831
July 2019 - August 2020	0.342	1.978	0.812
July 2019 - September 2020	-0.291	-1.709	0.931
July 2019 - May 2021	-0.718	-3.335	0.060
**July 2019 - June 2021**	**-1.226**	**-4.603**	**<0.001**

July 2019 - July 2021	-0.188	-0.922	1.000
July 2019 - August 2021	0.067	0.357	1.000
July 2019 - September 2021	-0.268	-1.468	0.981
August 2019 - September 2019	-0.059	-0.324	1.000
**August 2019 - April 2020**	**-0.879**	**-3.867**	**0.009**
**August 2019 - June 2020**	**-0.716**	**-3.859**	**0.010**
August 2019 - July 2020	0.008	0.048	1.000
**August 2019 - August 2020**	**0.674**	**4.008**	**0.005**
August 2019 - September 2020	0.040	0.244	1.000
August 2019 - May 2021	-0.387	-1.824	0.889
**August 2019 - June 2021**	**-0.895**	**-3.395**	**0.049**
August 2019 - July 2021	0.143	0.716	1.000

August 2019 - August 2021	0.399	2.174	0.684
August 2019 - September 2021	0.064	0.360	1.000
**September 2019 - April 2020**	**-0.819**	**-3.458**	**0.040**
September 2019 - June 2020	-0.657	-3.339	0.059
September 2019 - July 2020	0.067	0.384	1.000
**September 2019 - August 2020**	**0.733**	**4.057**	**0.004**
September 2019 - September 2020	0.100	0.561	1.000
September 2019 - May 2021	-0.327	-1.473	0.980
September 2019 - June 2021	-0.835	-3.076	0.126
September 2019 - July 2021	0.203	0.963	1.000
September 2019 - August 2021	0.458	2.360	0.545
September 2019 -	0.123	0.652	1.000

September 2021			
April 2020 - June 2020	0.162	0.708	1.000
**April 2020 - July 2020**	**0.886**	**4.187**	**0.003**
**April 2020 - August 2020**	**1.552**	**7.189**	**6.87293555401425 e-11**
**April 2020 - September 2020**	**0.919**	**4.318**	**0.001**
April 2020 - May 2021	0.492	1.962	0.821
April 2020 - June 2021	-0.016	-0.055	1.000
**April 2020 - July 2021**	**1.022**	**4.266**	**0.002**
**April 2020 - August 2021**	**1.278**	**5.733**	**<0.001**
**April 2020 - September 2021**	**0.943**	**4.291**	**0.002**
**June 2020 - July 2020**	**0.724**	**4.387**	**0.001**
**June 2020 - August 2020**	**1.390**	**8.157**	**<0.001**
**June 2020 - September 2020**	**0.757**	**4.521**	**0.001**
June 2020 - May 2021	0.330	1.551	0.968
June 2020 - June 2021	-0.178	-0.676	1.000

**June 2020 - July 2021**	**0.860**	**4.299**	**0.002**
**June 2020 - August 2021**	**1.115**	**6.146**	**<0.001**
**June 2020 - September 2021**	**0.780**	**4.414**	**0.001**
**July 2020 - August 2020**	**0.666**	**4.528**	**0.001**
July 2020 - September 2020	0.033	0.228	1.000
July 2020 - May 2021	-0.394	-2.022	0.786
**July 2020 - June 2021**	**-0.903**	**-3.606**	**0.025**
July 2020 - July 2021	0.136	0.748	1.000
July 2020 - August 2021	0.391	2.428	0.494
July 2020 - September 2021	0.056	0.361	1.000
**August 2020 - September 2020**	**-0.633**	**-4.233**	**0.002**
**August 2020 - May 2021**	**-1.060**	**-5.303**	**<0.001**
**August 2020 - June 2021**	**-1.568**	**-6.175**	**<0.001**

August 2020 - July 2021	-0.530	-2.849	0.221
August 2020 - August 2021	-0.275	-1.653	0.947
**August 2020 - September 2021**	**-0.610**	**-3.789**	**0.013**
September 2020 - May 2021	-0.427	-2.159	0.695
**September 2020 - June 2021**	**-0.935**	**-3.708**	**0.017**
September 2020 - July 2021	0.103	0.561	1.000
September 2020 - August 2021	0.359	2.207	0.660
September 2020 - September 2021	0.024	0.149	1.000
May 2021 - June 2021	-0.508	-1.879	0.864
May 2021 - July 2021	0.530	2.494	0.445
**May 2021 - August 2021**	**0.785**	**3.955**	**0.007**
May 2021 - September 2021	0.450	2.331	0.567

**June 2021 - July 2021**	**1.038**	**3.955**	**0.007**
**June 2021 - August 2021**	**1.294**	**5.148**	**<0.001**
**June 2021 - September 2021**	**0.959**	**3.880**	**0.009**
July 2021 - August 2021	0.256	1.390	0.988
July 2021 - September 2021	-0.079	-0.444	1.000
August 2021 - September 2021	-0.335	-2.111	0.728

Supplementary Information Table 5: Pairwise comparisons of the occurrence of infertile mother mites in single infested cells over the study period. Factors denoted in bold indicate significant differences between groups (p< 0.05; Tukey-Method adjusted for comparing 15 estimates and averaged over recapping status of cells).

Contrast	Estimate	Z	p
April 2019 - June 2019	0.611	1.453	0.982
April 2019 - July 2019	-0.204	-0.553	1.000
April 2019 - August 2019	-0.791	-2.255	0.624
**April 2019 - September 2019**	**-1.308**	**-3.705**	**0.017**
April 2019 - April 2020	-0.953	-2.427	0.494
April 2019 - June 2020	-0.408	-1.129	0.999

April 2019 - July 2020	-0.495	-1.450	0.983
April 2019 - August 2020	0.225	0.630	1.000
April 2019 - September 2020	-0.770	-2.269	0.614
**April 2019 - May 2021**	**-1.307**	**-3.523**	**0.033**
**April 2019 - June 2021**	**-1.415**	**-3.505**	**0.035**
April 2019 - July 2021	-0.488	-1.295	0.994
April 2019 - August 2021	-0.780	-2.232	0.641
April 2019 - September 2021	-0.583	-1.654	0.947
June 2019 - July 2019	-0.814	-2.345	0.556
**June 2019 - August 2019**	**-1.402**	**-4.235**	**0.002**
**June 2019 - September 2019**	**-1.919**	**-5.750**	**<0.001**
**June 2019 - April 2020**	**-1.564**	**-4.120**	**0.003**
June 2019 - June 2020	-1.018	-2.944	0.176
**June 2019 - July 2020**	**-1.106**	**-3.419**	**0.046**
June 2019 - August 2020	-0.385	-1.131	0.999

**June 2019 - September 2020**	**-1.380**	**-4.299**	**0.002**
**June 2019 - May 2021**	**-1.918**	**-5.389**	**<0.001**
**June 2019 - June 2021**	**-2.026**	**-5.196**	**<0.001**
June 2019 - July 2021	-1.099	-3.028	0.142
**June 2019 - August 2021**	**-1.391**	**-4.139**	**0.003**
**June 2019 - September 2021**	**-1.194**	**-3.524**	**0.032**
July 2019 - August 2019	-0.587	-2.338	0.561
**July 2019 - September 2019**	**-1.104**	**-4.345**	**0.001**
July 2019 - April 2020	-0.749	-2.392	0.521
July 2019 - June 2020	-0.204	-0.742	1.000
July 2019 - July 2020	-0.292	-1.198	0.997
July 2019 - August 2020	0.429	1.607	0.958
July 2019 - September 2020	-0.566	-2.342	0.559
**July 2019 - May 2021**	**-1.103**	**-3.869**	**0.009**
**July 2019 - June 2021**	**-1.212**	**-3.703**	**0.017**

July 2019 - July 2021	-0.285	-0.965	1.000
July 2019 - August 2021	-0.577	-2.190	0.672
July 2019 - September 2021	-0.380	-1.429	0.985
August 2019 - September 2019	-0.517	-2.348	0.554
August 2019 - April 2020	-0.162	-0.553	1.000
August 2019 - June 2020	0.383	1.527	0.972
August 2019 - July 2020	0.296	1.367	0.990
**August 2019 - August 2020**	**1.016**	**4.204**	**0.002**
August 2019 - September 2020	0.021	0.101	1.000
August 2019 - May 2021	-0.516	-1.961	0.821
August 2019 - June 2021	-0.624	-2.026	0.783
August 2019 - July 2021	0.303	1.110	0.999

August 2019 - August 2021	0.011	0.045	1.000
August 2019 - September 2021	0.208	0.863	1.000
September 2019 - April 2020	0.355	1.207	0.997
**September 2019 - June 2020**	**0.900**	**3.563**	**0.028**
**September 2019 - July 2020**	**0.813**	**3.720**	**0.016**
**September 2019 - August 2020**	**1.533**	**6.300**	**<0.001**
September 2019 - September 2020	0.539	2.504	0.437
September 2019 - May 2021	0.001	0.004	1.000
September 2019 - June 2021	-0.107	-0.346	1.000
September 2019 - July 2021	0.820	2.991	0.157
September 2019 - August 2021	0.528	2.221	0.650
September 2019 -	0.725	2.998	0.154

September 2021			
April 2020 - June 2020	0.545	1.812	0.894
April 2020 - July 2020	0.458	1.669	0.943
**April 2020 - August 2020**	**1.178**	**4.012**	**0.005**
April 2020 - September 2020	0.184	0.681	1.000
April 2020 - May 2021	-0.354	-1.143	0.998
April 2020 - June 2021	-0.462	-1.321	0.993
April 2020 - July 2021	0.465	1.472	0.980
April 2020 - August 2021	0.173	0.615	1.000
April 2020 - September 2021	0.370	1.292	0.994
June 2020 - July 2020	-0.088	-0.381	1.000
June 2020 - August 2020	0.633	2.500	0.440
June 2020 - September 2020	-0.362	-1.604	0.958
June 2020 - May 2021	-0.899	-3.294	0.068
June 2020 - June 2021	-1.008	-3.176	0.095

June 2020 - July 2021	-0.080	-0.286	1.000
June 2020 - August 2021	-0.372	-1.521	0.973
June 2020 - September 2021	-0.176	-0.703	1.000
July 2020 - August 2020	0.721	3.252	0.077
July 2020 - September 2020	-0.274	-1.452	0.982
July 2020 - May 2021	-0.812	-3.324	0.062
July 2020 - June 2021	-0.920	-3.139	0.106
July 2020 - July 2021	0.007	0.029	1.000
July 2020 - August 2021	-0.285	-1.338	0.992
July 2020 - September 2021	-0.088	-0.404	1.000
**August 2020 - September 2020**	**-0.995**	**-4.590**	**<0.001**
**August 2020 - May 2021**	**-1.532**	**-5.741**	**<0.001**
**August 2020 - June 2021**	**-1.640**	**-5.257**	**<0.001**

August 2020 - July 2021	-0.713	-2.595	0.373
**August 2020 - August 2021**	**-1.005**	**-4.240**	**0.002**
August 2020 - September 2021	-0.808	-3.341	0.058
September 2020 - May 2021	-0.537	-2.224	0.647
September 2020 - June 2021	-0.646	-2.221	0.650
September 2020 - July 2021	0.281	1.126	0.999
September 2020 - August 2021	-0.011	-0.052	1.000
September 2020 - September 2021	0.186	0.875	1.000
May 2021 - June 2021	-0.108	-0.371	1.000
May 2021 - July 2021	0.819	3.142	0.105
May 2021 - August 2021	0.527	2.316	0.578
May 2021 - September 2021	0.724	3.138	0.106

June 2021 - July 2021	0.927	3.053	0.134
June 2021 - August 2021	0.635	2.306	0.586
June 2021 - September 2021	0.832	2.996	0.155
July 2021 - August 2021	-0.292	-1.237	0.996
July 2021 - September 2021	-0.095	-0.397	1.000
August 2021 - September 2021	0.197	0.998	1.000

Supplementary Information Table 6: Pairwise comparisons of the occurrence of delayed reproduction in single infested cells over the study period. Factors denoted in bold indicate significant differences between groups ($p < 0.05$; Tukey-Method adjusted for comparing 15 estimates and averaged over recapping status of cells).

Contrast	Estimate	Z	p
April 2019 - June 2019	-0.597	-1.565	0.966
April 2019 - July 2019	-0.757	-2.062	0.760
April 2019 - August 2019	-0.400	-1.060	0.999
April 2019 - September 2019	0.013	0.031	1.000
**April 2019 - April 2020**	**-1.562**	**-4.043**	**0.005**
**April 2019 - June 2020**	**-1.727**	**-4.808**	**<0.001**

April 2019 - July 2020	-0.948	-2.678	0.319
April 2019 - August 2020	-0.094	-0.253	1.000
April 2019 - September 2020	-0.651	-1.802	0.898
April 2019 - May 2021	-0.635	-1.586	0.962
April 2019 - June 2021	-1.336	-3.238	0.080
April 2019 - July 2021	-0.760	-2.002	0.798
April 2019 - August 2021	-0.214	-0.565	1.000
April 2019 - September 2021	-0.796	-2.198	0.666
June 2019 - July 2019	-0.160	-0.654	1.000
June 2019 - August 2019	0.197	0.762	1.000
June 2019 - September 2019	0.610	1.990	0.805
**June 2019 - April 2020**	**-0.965**	**-3.496**	**0.036**
**June 2019 - June 2020**	**-1.130**	**-4.789**	**<0.001**
June 2019 - July 2020	-0.351	-1.545	0.970
June 2019 - August 2020	0.503	2.007	0.795

June 2019 - September 2020	-0.054	-0.226	1.000
June 2019 - May 2021	-0.038	-0.128	1.000
June 2019 - June 2021	-0.739	-2.379	0.531
June 2019 - July 2021	-0.163	-0.613	1.000
June 2019 - August 2021	0.383	1.435	0.984
June 2019 - September 2021	-0.199	-0.824	1.000
July 2019 - August 2019	0.357	1.526	0.973
July 2019 - September 2019	0.769	2.690	0.311
July 2019 - April 2020	-0.806	-3.151	0.102
**July 2019 - June 2020**	**-0.970**	**-4.591**	**<0.001**
July 2019 - July 2020	-0.191	-0.955	1.000
July 2019 - August 2020	0.663	2.918	0.188
July 2019 - September 2020	0.106	0.493	1.000
July 2019 - May 2021	0.122	0.445	1.000
July 2019 - June 2021	-0.580	-1.988	0.806

July 2019 - July 2021	-0.004	-0.016	1.000
July 2019 - August 2021	0.542	2.196	0.668
July 2019 - September 2021	-0.040	-0.182	1.000
August 2019 - September 2019	0.412	1.382	0.989
**August 2019 - April 2020**	**-1.163**	**-4.312**	**0.002**
**August 2019 - June 2020**	**-1.327**	**-5.819**	**<0.001**
August 2019 - July 2020	-0.548	-2.513	0.431
August 2019 - August 2020	0.306	1.259	0.996
August 2019 - September 2020	-0.251	-1.088	0.999
August 2019 - May 2021	-0.235	-0.818	1.000
August 2019 - June 2021	-0.937	-3.080	0.124
August 2019 - July 2021	-0.361	-1.389	0.988

August 2019 - August 2021	0.185	0.710	1.000
August 2019 - September 2021	-0.397	-1.685	0.938
**September 2019 - April 2020**	**-1.575**	**-5.020**	**<0.001**
**September 2019 - June 2020**	**-1.739**	**-6.235**	**<0.001**
**September 2019 - July 2020**	**-0.960**	**-3.540**	**0.031**
September 2019 - August 2020	-0.106	-0.364	1.000
September 2019 - September 2020	-0.664	-2.361	0.545
September 2019 - May 2021	-0.647	-1.965	0.819
**September 2019 - June 2021**	**-1.349**	**-3.916**	**0.008**
September 2019 - July 2021	-0.773	-2.533	0.416
September 2019 - August 2021	-0.227	-0.743	1.000
September 2019 -	-0.809	-2.851	0.220

September 2021			
April 2020 - June 2020	-0.164	-0.683	1.000
April 2020 - July 2020	0.615	2.637	0.345
**April 2020 - August 2020**	**1.469**	**5.751**	**<0.001**
**April 2020 - September 2020**	**0.911**	**3.760**	**0.014**
April 2020 - May 2021	0.928	3.104	0.117
April 2020 - June 2021	0.226	0.716	1.000
April 2020 - July 2021	0.802	2.967	0.167
**April 2020 - August 2021**	**1.348**	**5.051**	**<0.001**
April 2020 - September 2021	0.766	3.137	0.106
**June 2020 - July 2020**	**0.779**	**4.219**	**0.002**
**June 2020 - August 2020**	**1.633**	**7.690**	**<0.001**
**June 2020 - September 2020**	**1.076**	**5.461**	**<0.001**
**June 2020 - May 2021**	**1.092**	**4.153**	**0.003**
June 2020 - June 2021	0.390	1.385	0.989

**June 2020 - July 2021**	**0.966**	**4.192**	**0.003**
**June 2020 - August 2021**	**1.513**	**6.643**	**<0.001**
**June 2020 - September 2021**	**0.930**	**4.654**	**<0.001**
**July 2020 - August 2020**	**0.854**	**4.203**	**0.002**
July 2020 - September 2020	0.297	1.584	0.962
July 2020 - May 2021	0.313	1.226	0.997
July 2020 - June 2021	-0.388	-1.413	0.986
July 2020 - July 2021	0.187	0.844	1.000
July 2020 - August 2021	0.734	3.328	0.061
July 2020 - September 2021	0.151	0.794	1.000
August 2020 - September 2020	-0.557	-2.595	0.373
August 2020 - May 2021	-0.541	-1.959	0.823
**August 2020 - June 2021**	**-1.243**	**-4.219**	**0.002**

August 2020 - July 2021	-0.667	-2.714	0.296
August 2020 - August 2021	-0.121	-0.496	1.000
August 2020 - September 2021	-0.703	-3.235	0.080
September 2020 - May 2021	0.016	0.061	1.000
September 2020 - June 2021	-0.685	-2.413	0.505
September 2020 - July 2021	-0.110	-0.471	1.000
September 2020 - August 2021	0.437	1.904	0.852
September 2020 - September 2021	-0.145	-0.719	1.000
May 2021 - June 2021	-0.702	-2.131	0.714
May 2021 - July 2021	-0.126	-0.437	1.000
May 2021 - August 2021	0.421	1.466	0.981
May 2021 - September 2021	-0.162	-0.611	1.000

June 2021 - July 2021	0.576	1.889	0.859
**June 2021 - August 2021**	**1.122**	**3.686**	**0.019**
June 2021 - September 2021	0.540	1.905	0.851
July 2021 - August 2021	0.546	2.132	0.714
July 2021 - September 2021	-0.036	-0.155	1.000
August 2021 - September 2021	-0.582	-2.557	0.400

Supplementary Information Table 7: Pairwise comparisons of the occurrence of missing males in single infested cells over the study period. Factors denoted in bold indicate significant differences between groups (p< 0.05; Tukey-Method adjusted for comparing 15 estimates and averaged over recapping status of cells).

Contrast	Estimate	Z	p
April 2019 - June 2019	0.261	0.549	1.000
April 2019 - July 2019	0.849	1.720	0.927
April 2019 - August 2019	-0.151	-0.351	1.000
April 2019 - September 2019	0.305	0.638	1.000
April 2019 - April 2020	-0.456	-0.943	1.000
April 2019 - June 2020	-0.320	-0.733	1.000

April 2019 - July 2020	0.346	0.795	1.000
April 2019 - August 2020	-0.136	-0.329	1.000
April 2019 - September 2020	0.450	1.003	1.000
April 2019 - May 2021	0.307	0.586	1.000
April 2019 - June 2021	0.706	1.009	1.000
April 2019 - July 2021	0.397	0.798	1.000
April 2019 - August 2021	2.442	3.045	0.137
April 2019 - September 2021	0.323	0.719	1.000
June 2019 - July 2019	0.588	1.332	0.992
June 2019 - August 2019	-0.412	-1.110	0.999
June 2019 - September 2019	0.044	0.103	1.000
June 2019 - April 2020	-0.717	-1.642	0.950
June 2019 - June 2020	-0.581	-1.513	0.975
June 2019 - July 2020	0.085	0.223	1.000
June 2019 - August 2020	-0.397	-1.117	0.999

June 2019 - September 2020	0.189	0.476	1.000
June 2019 - May 2021	0.046	0.097	1.000
June 2019 - June 2021	0.445	0.669	1.000
June 2019 - July 2021	0.136	0.302	1.000
June 2019 - August 2021	2.181	2.814	0.239
June 2019 - September 2021	0.062	0.157	1.000
July 2019 - August 2019	-1.000	-2.585	0.380
July 2019 - September 2019	-0.544	-1.231	0.997
July 2019 - April 2020	-1.306	-2.873	0.209
July 2019 - June 2020	-1.169	-2.905	0.194
July 2019 - July 2020	-0.504	-1.267	0.995
July 2019 - August 2020	-0.985	-2.632	0.348
July 2019 - September 2020	-0.400	-0.963	1.000
July 2019 - May 2021	-0.542	-1.099	0.999
July 2019 - June 2021	-0.143	-0.211	1.000

July 2019 - July 2021	-0.453	-0.971	1.000
July 2019 - August 2021	1.593	2.025	0.783
July 2019 - September 2021	-0.526	-1.260	0.996
August 2019 - September 2019	0.456	1.251	0.996
August 2019 - April 2020	-0.306	-0.802	1.000
August 2019 - June 2020	-0.169	-0.534	1.000
August 2019 - July 2020	0.496	1.594	0.960
August 2019 - August 2020	0.015	0.053	1.000
August 2019 - September 2020	0.600	1.808	0.895
August 2019 - May 2021	0.458	1.065	0.999
August 2019 - June 2021	0.857	1.354	0.991
August 2019 - July 2021	0.547	1.382	0.989

**August 2019 - August 2021**	**2.593**	**3.475**	**0.038**
August 2019 - September 2021	0.474	1.406	0.987
September 2019 - April 2020	-0.762	-1.749	0.918
September 2019 - June 2020	-0.625	-1.643	0.949
September 2019 - July 2020	0.040	0.107	1.000
September 2019 - August 2020	-0.441	-1.259	0.996
September 2019 - September 2020	0.144	0.368	1.000
September 2019 - May 2021	0.002	0.004	1.000
September 2019 - June 2021	0.401	0.600	1.000
September 2019 - July 2021	0.091	0.204	1.000
September 2019 - August 2021	2.137	2.757	0.270
September 2019 -	0.018	0.045	1.000

September 2021			
April 2020 - June 2020	0.136	0.353	1.000
April 2020 - July 2020	0.802	2.096	0.738
April 2020 - August 2020	0.321	0.901	1.000
April 2020 - September 2020	0.906	2.286	0.601
April 2020 - May 2021	0.763	1.579	0.963
April 2020 - June 2021	1.163	1.733	0.923
April 2020 - July 2021	0.853	1.888	0.860
**April 2020 - August 2021**	**2.899**	**3.747**	**0.015**
April 2020 - September 2021	0.779	1.957	0.824
June 2020 - July 2020	0.666	2.072	0.754
June 2020 - August 2020	0.184	0.636	1.000
June 2020 - September 2020	0.770	2.271	0.613
June 2020 - May 2021	0.627	1.432	0.985
June 2020 - June 2021	1.026	1.607	0.958

June 2020 - July 2021	0.717	1.778	0.907
**June 2020 - August 2021**	**2.762**	**3.695**	**0.018**
June 2020 - September 2021	0.643	1.873	0.867
July 2020 - August 2020	-0.481	-1.689	0.937
July 2020 - September 2020	0.104	0.311	1.000
July 2020 - May 2021	-0.038	-0.088	1.000
July 2020 - June 2021	0.361	0.566	1.000
July 2020 - July 2021	0.051	0.128	1.000
July 2020 - August 2021	2.097	2.809	0.241
July 2020 - September 2021	-0.022	-0.066	1.000
August 2020 - September 2020	0.585	1.922	0.842
August 2020 - May 2021	0.443	1.073	0.999
August 2020 - June 2021	0.842	1.354	0.991

August 2020 - July 2021	0.532	1.421	0.986
**August 2020 - August 2021**	**2.578**	**3.519**	**0.033**
August 2020 - September 2021	0.459	1.481	0.979
September 2020 - May 2021	-0.143	-0.318	1.000
September 2020 - June 2021	0.257	0.397	1.000
September 2020 - July 2021	-0.053	-0.128	1.000
September 2020 - August 2021	1.993	2.647	0.338
September 2020 - September 2021	-0.127	-0.356	1.000
May 2021 - June 2021	0.399	0.576	1.000
May 2021 - July 2021	0.089	0.183	1.000
May 2021 - August 2021	2.135	2.671	0.323
May 2021 - September 2021	0.016	0.036	1.000

June 2021 - July 2021	-0.310	-0.459	1.000
June 2021 - August 2021	1.736	1.878	0.865
June 2021 - September 2021	-0.383	-0.597	1.000
July 2021 - August 2021	2.046	2.621	0.355
July 2021 - September 2021	-0.074	-0.179	1.000
August 2021 - September 2021	-2.119	-2.822	0.235

Supplementary Information Table 8: Pairwise comparisons of the occurrence of REC in single infested cells over the study period. Factors denoted in bold indicate significant differences between groups (p< 0.05; Tukey-Method adjusted for comparing 15 estimates and averaged over reproductive status of cells).

Contrast	Estimate	Z	p
April 2019 - June 2019	-0.549	-2.299	0.591
**April 2019 - July 2019**	**-1.735**	**-7.048**	**<0.001**
April 2019 - August 2019	-1.045	-4.363	0.001
April 2019 - September 2019	-0.490	-1.948	0.829
April 2019 - April 2020	0.950	3.104	0.116
April 2019 - June 2020	0.333	1.364	0.990

April 2019 - July 2020	-0.040	-0.179	1.000
April 2019 - August 2020	0.238	1.071	0.999
April 2019 - September 2020	0.665	2.874	0.208
April 2019 - May 2021	-0.754	-2.611	0.362
April 2019 - June 2021	-0.559	-1.666	0.943
April 2019 - July 2021	0.106	0.394	1.000
**April 2019 - August 2021**	**1.513**	**5.636**	**<0.001**
April 2019 - September 2021	0.427	1.714	0.929
**June 2019 - July 2019**	**-1.186**	**-5.782**	**<0.001**
June 2019 - August 2019	-0.496	-2.502	0.439
June 2019 - September 2019	0.059	0.275	1.000
**June 2019 - April 2020**	**1.499**	**5.332**	**<0.001**
June 2019 - June 2020	0.883	4.205	0.002
June 2019 - July 2020	0.509	2.760	0.269
June 2019 - August 2020	0.788	4.300	0.002

**June 2019 - September 2020**	**1.214**	**6.330**	**<0.001**
June 2019 - May 2021	-0.205	-0.781	1.000
June 2019 - June 2021	-0.010	-0.030	1.000
June 2019 - July 2021	0.656	2.713	0.297
**June 2019 - August 2021**	**2.062**	**8.530**	**<0.001**
**June 2019 - September 2021**	**0.976**	**4.453**	**0.001**
**July 2019 - August 2019**	**0.690**	**3.453**	**0.041**
**July 2019 - September 2019**	**1.245**	**5.799**	**<0.001**
**July 2019 - April 2020**	**2.685**	**9.527**	**<0.001**
**July 2019 - June 2020**	**2.068**	**9.761**	**<0.001**
**July 2019 - July 2020**	**1.695**	**9.119**	**<0.001**
**July 2019 - August 2020**	**1.973**	**10.665**	**<0.001**
**July 2019 - September 2020**	**2.400**	**12.408**	**<0.001**
**July 2019 - May 2021**	**0.981**	**3.732**	**0.016**
**July 2019 - June 2021**	**1.176**	**3.753**	**0.015**

**July 2019 - July 2021**	**1.841**	**7.562**	**<0.001**
**July 2019 - August 2021**	**3.248**	**13.323**	**<0.001**
**July 2019 - September 2021**	**2.162**	**9.771**	**<0.001**
August 2019 - September 2019	0.554	2.773	0.261
**August 2019 - April 2020**	**1.994**	**7.228**	**<0.001**
**August 2019 - June 2020**	**1.378**	**6.780**	**<0.001**
**August 2019 - July 2020**	**1.005**	**5.733**	**<0.001**
**August 2019 - August 2020**	**1.283**	**7.360**	**<0.001**
**August 2019 - September 2020**	**1.710**	**9.320**	**<0.001**
August 2019 - May 2021	0.291	1.133	0.999
August 2019 - June 2021	0.486	1.576	0.964
**August 2019 - July 2021**	**1.151**	**4.864**	**<0.001**

**August 2019 - August 2021**	**2.558**	**10.792**	**<0.001**
**August 2019 - September 2021**	**1.472**	**6.880**	**<0.001**
**September 2019 - April 2020**	**1.440**	**5.062**	**<0.001**
**September 2019 - June 2020**	**0.824**	**3.819**	**0.011**
September 2019 - July 2020	0.450	2.374	0.534
**September 2019 - August 2020**	**0.729**	**3.865**	**0.010**
**September 2019 - September 2020**	**1.155**	**5.874**	**<0.001**
September 2019 - May 2021	-0.264	-0.988	1.000
September 2019 - June 2021	-0.068	-0.216	1.000
September 2019 - July 2021	0.597	2.413	0.505
**September 2019 - August 2021**	**2.003**	**8.101**	**<0.001**
**September 2019 -**	**0.917**	**4.066**	**0.004**

**September 2021**			
April 2020 - June 2020	-0.616	-2.266	0.616
**April 2020 - July 2020**	**-0.990**	**-3.907**	**0.008**
April 2020 - August 2020	-0.711	-2.808	0.242
April 2020 - September 2020	-0.285	-1.098	0.999
**April 2020 - May 2021**	**-1.704**	**-5.511**	**<0.001**
**April 2020 - June 2021**	**-1.508**	**-4.277**	**0.002**
April 2020 - July 2021	-0.843	-2.895	0.198
April 2020 - August 2021	0.563	1.937	0.834
April 2020 - September 2021	-0.523	-1.923	0.842
June 2020 - July 2020	-0.374	-2.139	0.709
June 2020 - August 2020	-0.095	-0.543	1.000
June 2020 - September 2020	0.332	1.808	0.896
**June 2020 - May 2021**	**-1.087**	**-4.326**	**0.001**
June 2020 - June 2021	-0.892	-2.937	0.179

June 2020 - July 2021	-0.227	-0.984	1.000
**June 2020 - August 2021**	**1.179**	**5.087**	**<0.001**
June 2020 - September 2021	0.093	0.450	1.000
July 2020 - August 2020	0.279	1.968	0.817
**July 2020 - September 2020**	**0.705**	**4.654**	**<0.001**
July 2020 - May 2021	-0.714	-3.086	0.122
July 2020 - June 2021	-0.519	-1.801	0.898
July 2020 - July 2021	0.147	0.705	1.000
**July 2020 - August 2021**	**1.553**	**7.435**	**<0.001**
July 2020 - September 2021	0.467	2.566	0.393
August 2020 - September 2020	0.427	2.822	0.235
**August 2020 - May 2021**	**-0.992**	**-4.293**	**0.002**
August 2020 - June 2021	-0.797	-2.765	0.266

August 2020 - July 2021	-0.132	-0.638	1.000
**August 2020 - August 2021**	**1.274**	**6.136**	**<0.001**
August 2020 - September 2021	0.188	1.041	0.999
**September 2020 - May 2021**	**-1.419**	**-5.951**	**<0.001**
**September 2020 - June 2021**	**-1.224**	**-4.167**	**0.003**
September 2020 - July 2021	-0.559	-2.591	0.376
**September 2020 - August 2021**	**0.848**	**3.912**	**0.008**
September 2020 - September 2021	-0.238	-1.248	0.996
May 2021 - June 2021	0.195	0.669	1.000
**May 2021 - July 2021**	**0.860**	**3.689**	**0.018**
**May 2021 - August 2021**	**2.267**	**9.381**	**<0.001**
**May 2021 - September 2021**	**1.181**	**5.435**	**<0.001**

June 2021 - July 2021	0.665	2.350	0.553
**June 2021 - August 2021**	**2.072**	**7.132**	**<0.001**
**June 2021 - September 2021**	**0.986**	**3.646**	**0.021**
**July 2021 - August 2021**	**1.406**	**6.473**	**<0.001**
July 2021 - September 2021	0.320	1.668	0.943
**August 2021 - September 2021**	**-1.086**	**-5.543**	**<0.001**

Supplementary Information Table 9: Schedule of picture trials (7 pictures per trial) and subsequent sampling of brood combs for later investigations. Picture intervals partly match with brood termination protocols of OECD guideline 75 [1]. Note that brood fixation day (BFD) is the day of egg laying according to [1].

**Picture No.**	**Days relative to capping (± 1 day)**	**Expected brood stadium**	**OECD 75 [1]**
1	-2	Old larvae	-
2	0	Sealed brood	-
3	2	Sealed brood	BFD +10
4	4	Sealed brood	-
5	6	Sealed brood	-
6	8	Sealed brood	BFD +16
7 sampling of brood combs	10	Sealed brood	-

References

1. OECD (2014), *Guidance Document on the Honey Bee (Apis Mellifera L.) Brood test Under Semi-field Conditions*, OECD Series on Testing and Assessment, No. 75, OECD Publishing, Paris, https://doi.org/10.1787/9789264085510-en
